# Novel Adiponectin Variants Identified in Type 2 Diabetic Patients Reveal Multimerization and Secretion Defects

**DOI:** 10.1371/journal.pone.0026792

**Published:** 2011-10-26

**Authors:** Prapaporn Jungtrakoon, Nattachet Plengvidhya, Watip Tangjittipokin, Sarin Chimnaronk, Wanisa Salaemae, Nalinee Chongjaroen, Kanjana Chanprasert, Jatuporn Sujjitjoon, Chatchawan Srisawat, Pa-thai Yenchitsomanus

**Affiliations:** 1 Department of Immunology, Faculty of Medicine Siriraj Hospital, Mahidol University, Bangkok, Thailand; 2 Division of Endocrinology and Metabolism, Department of Medicine, Faculty of Medicine Siriraj Hospital, Mahidol University, Bangkok, Thailand; 3 Institute of Molecular Biosciences, Mahidol University, Nakhonpathom, Thailand; 4 Department of Biochemistry, Faculty of Medicine Siriraj Hospital, Mahidol University, Bangkok, Thailand; 5 Division of Medical Molecular Biology, Department of Research and Development, Faculty of Medicine Siriraj Hospital, Mahidol University, Bangkok, Thailand; University of Tor Vergata, Italy

## Abstract

*ADIPOQ*, encoding adiponectin, is a candidate gene for type 2 diabetes (T2D) identified by genome-wide linkage analyses with supporting evidence showing the protein function in sensitizing insulin actions. In an endeavor to characterize candidate genes causing T2D in Thai patients, we identified 10 novel *ADIPOQ* variations, several of which were non-synonymous variations observed only in the patients. To examine the impact of these non-synonymous variations on adiponectin structure and biochemical characteristics, we conducted a structural analysis of the wild-type and variant proteins by *in silico* modeling and further characterized biochemical properties of the variants with predicted structural abnormalities from the modeling by molecular and biochemical studies. The recombinant plasmids containing wild-type and variant *ADIPOQ* cDNAs derived from the variations identified by our study (R55H, R112H, and R131H) and previous work (G90S and R112C) were constructed and transiently expressed and co-expressed in cultured HEK293T cells to investigate their oligomerization, interaction, and secretion. We found that the novel R55H variant impaired protein multimerization but it did not exert the effect over the co-expressed wild-type protein while novel R131H variant impaired protein secretion and also affected the co-expressed wild-type protein in a dominant negative fashion. The R131H variant could traffic from the endoplasmic reticulum to the Golgi, trans-Golgi network, and early endosome but could not be secreted. The R131H variant was likely to be degraded through the lysosomal system and inhibition of its degradation rescued the variant protein from secretion defect. We have shown the possibility of using *in silico* modeling for predicting the effect of amino acid substitution on adiponectin oligomerization. This is also the first report that demonstrates a dominant negative effect of the R131H variant on protein secretion and the possibility of using protein degradation inhibitors as therapeutic agents in the patients carrying adiponectin variants with secretion defect.

## Introduction

Type 2 diabetes (T2D) is a multifactorial disorder in which the interactions between susceptible genetic and particular environmental factors are attributable to its pathogenesis. After the breakthrough of microarray technologies, several T2D-predisposing genetic variations have been identified by a genome-wide association study (GWAS), following the concept of common disease-common variant (CDCV) hypothesis [1]. However, it has now been realized that those common variations conferred a small risk and most of their pathogenic effects are elusive. The trend in searching for genetic components of T2D is thus shifted towards the common disease-rare variant (CDRV) hypothesis [2]. The rare variants with risk can be detected by exome [3] or whole genome sequencing [4] but the methods based on candidate gene analysis are still practical in terms of their simplicity and cost effectiveness.

*ADIPOQ*, encoding adiponectin, is a candidate gene for T2D as evidenced from both genome-wide linkage and its biological functions in sensitizing insulin actions. This gene is located on chromosome 3q27, the region where the gene responsible for T2D was mapped [5], [6]. Adiponectin is exclusively secreted from adipose tissue as three classes of oligomeric forms, including trimers, hexamers and high molecular weight (HMW) multimers [7]. Adiponectin is classified as a member of adipocytokines that facilitates insulin actions in peripheral tissues by activating AMP kinase and p38 MAPK [8]–[10]. Each adiponectin oligomer exerts distinct levels of insulin sensitizing effects and HMW multimer is the major active form of adiponectin [7], [11], [12].

Although none of GWAS has revealed *ADIPOQ* as T2D susceptible locus, the role of *ADIPOQ* in T2D development cannot be excluded since *ADIPOQ* rare variants were not probed by those studies. Various common *ADIPOQ* variants have been reported to be associated with plasma adiponectin levels, but none of them was consistently associated with T2D [13], [14]. By contrast, rare adiponectin variants leading to oligomerization and secretion defects have been found to be associated with hypoadiponectinemia and diabetic phenotypes [15]. It has been suggested that rare functional variants may have stronger effects than the slight changes in adiponectin levels induced by common variants [14].

We are interested in identifying functional *ADIPOQ* variants in Thai patients with T2D. Many novel variations were identified and interestingly several variations resulted in amino acid substitutions in adiponectin proteins. To initially predict the effect of these amino acid changes on the protein structure, we employed *in silico* modeling and mutagenesis for analysis to compare the structures of wild-type and variant adiponectins. Then, the wild-type and variant adiponectins with predicted structural abnormalities were further studied by molecular cloning and protein expression in cultured cells. The expressed proteins were analyzed for their oligomerization, interaction, and secretion. In addition, sub-cellular localization, degradation pathway and inhibition of the variant proteins were investigated. The results of this study will provide more insight into the molecular defect of adiponectin underlying T2D pathogenesis and a potential therapeutic approach based on a targeted molecule and specific molecular mechanism.

## Results

### Identification of *ADIPOQ* variations

**Figure 1:**
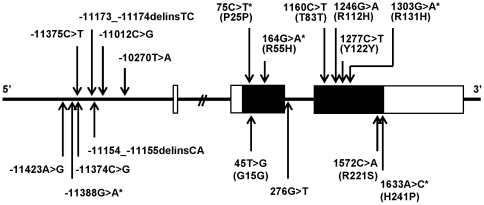
*ADIPOQ* structure. Three boxes represent 3 exons of *ADIPOQ*. The coding regions are indicated as filled boxes. The positions of 18 variations identified in this study are located. The variations in upper panel are novel and those in the lower panel were reported. The variations which were indentified in the patients with T2D but not in non-diabetic individuals are indicated with asterisks.

**Figure 2:**
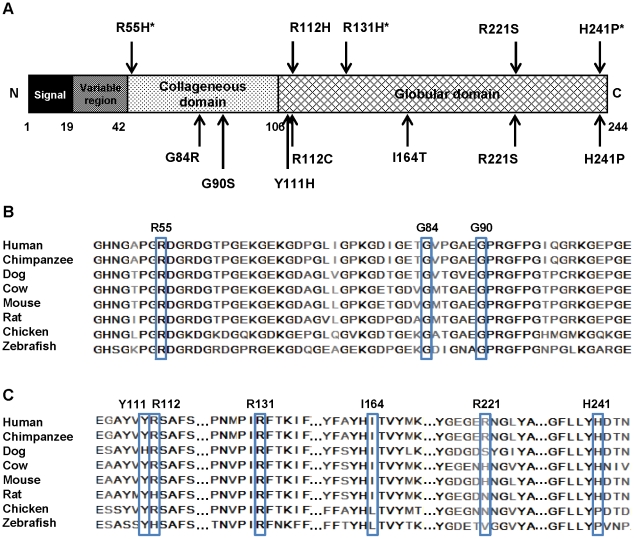
Adiponectin protein structure and amino acid alignment. A: Adiponectin protein is divided in to signal sequence, variable region, collageneous domain and globular domain. The upper panel indicates variants identified in this study. Those identified only in T2D patients are indicated with asterisks. The lower panel represents the variants that have been previously reported. B and C: Multiple alignments of adiponectin protein from different species using BioEdit Sequence Alignment Editor. The positions of amino acid substitutions are located. B: Variations located in collageneous domain. C: Variations located in globular domain.

A total of 18 variations (Figure 1 and Table S1) were identified in the studied subjects (Table 1). Ten of them are novel (Figure S1, NCBI SS#: 120239896–120239900 and 120246102–120246106). Five rare non-synonymous variations resulted in amino acid substitutions including R55H, R112H, R221S, R131H and H241P (Figure 2A). R55H, R131H, H241P, and two other variations (−11388G>A and P25P) were not observed in 210 non-diabetic controls, suggesting their roles in the development of T2D. By using computational analyses, −11388G>A is not located in any consensus sequence of transcription factor binding sites and P25P resided in neither cryptic splice site nor exonic splicing enhancer (ESE) (data not shown). Therefore, these two variations were not explored further in this study. Since non-synonymous variations are more likely to cause the disease, they were focused on in this study.

**Table 1:** Amino acid substitutions in collagenous and globular domains of adiponectins and their effects.

	Amino acid residue				Formation of oligomer			Phenotype		
Domain	Human	Other species	Amino acid substitution	Effect on local structure	Trimer	Hexamer	HMW	Diabetes	Hypoadiponectinemia	Population
Collagenous	R55	R	R55H	ND	Yes	Yes	No	Yes	ND	Thai
	G84	G	G84R	ND	Yes	Yes	No	Yes	Yes	Japanese [33], French [34]
	G90	G	G90S	ND	Yes	Yes	No	Yes	Yes	French [34]
Globular	Y111	Y and H	Y111H	ND	Yes	Yes	Yes	Yes	Yes	French [34]
	R112	R and H	R112C	Yes	No	No	No	Yes	Yes	Japanese [35]
			R112H	ND	No	No	No	No	ND	Thai
	R131	R	R131H	Yes	No	No	No	Yes	ND	Thai
	I164	I and L	I164T	Yes	No	No	No	Yes	Yes	Japanese [35], Japanese [33]
	R221	R, S, H, N, and V	R221S	ND	Yes	Yes	Yes	No	No	Japanese [35], Japanese [33], Thai
	H241	H and P	H241P	ND	Yes	Yes	Yes	No	No	Japanese [35], Japanese [33], Thai

ND stands for not determined.

### Amino acid substitutions, structural alterations and oligomerization of adiponectin

Seven adiponectin variants identified in other ethnic groups including G84R, G90S, Y111H, R112C, I164T, R221S, and H241P were previously characterized for their effect on protein oligomerization (Table 1) [15]. We analyzed all the reported variants and those identified in our study by amino acid sequence alignment (Figures 2B and 2C). It was observed that substitutions in the highly conserved amino acids (G84R, G90S, R112C, and I164T) affect protein oligomerization (Table 1) whereas substitutions at the less conserved positions (Y111H, R221S, and H241P) do not disturb formation of adiponectin oligomers (Table 1). However, the analyses of local structural alterations attributable to these amino acid substitutions in relation to their molecular defects by *in silico* modeling have not previously been conducted. To analyze this relationship, we compared the molecular structures of the previously reported variants and those identified in our study with that of the wild-type protein by *in silico* modeling and mutagenesis.

**Figure 3:**
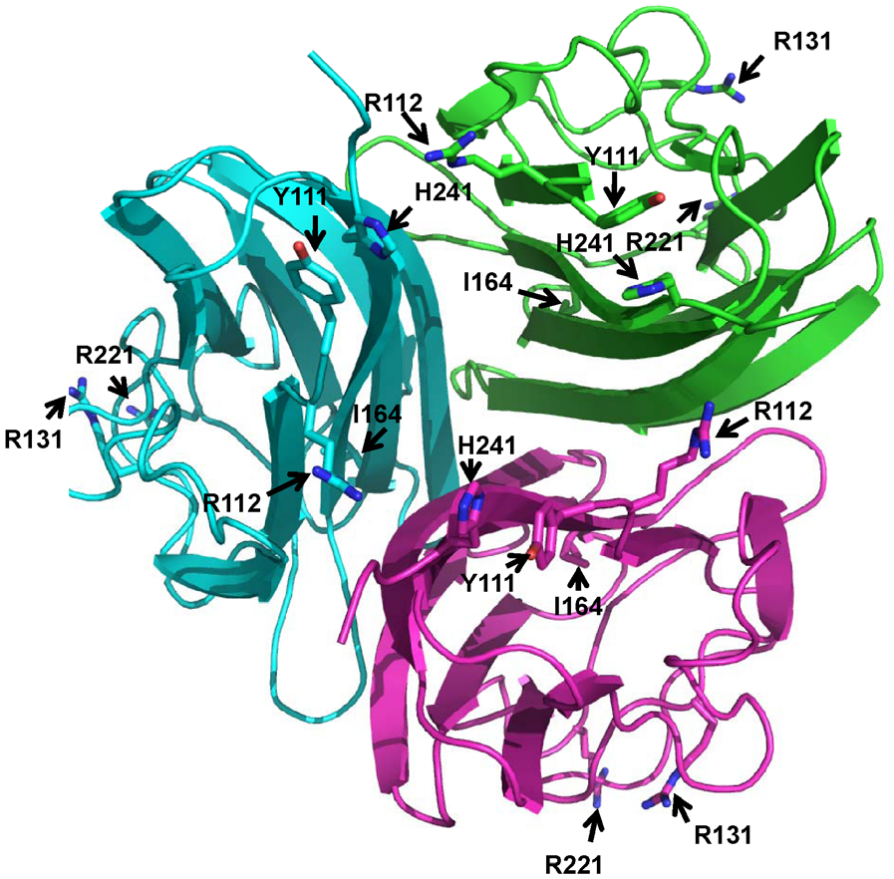
Three dimensional structure of modeled human adiponectin globular domain and positions of amino acid variations. Human adiponectin was constructed by homology modeling using mouse adiponectin [16] as a template. Each monomer of the adiponectin trimer structure is shown in different color. The positions of amino acids being substituted are indicated with arrows.

**Figure 4:**
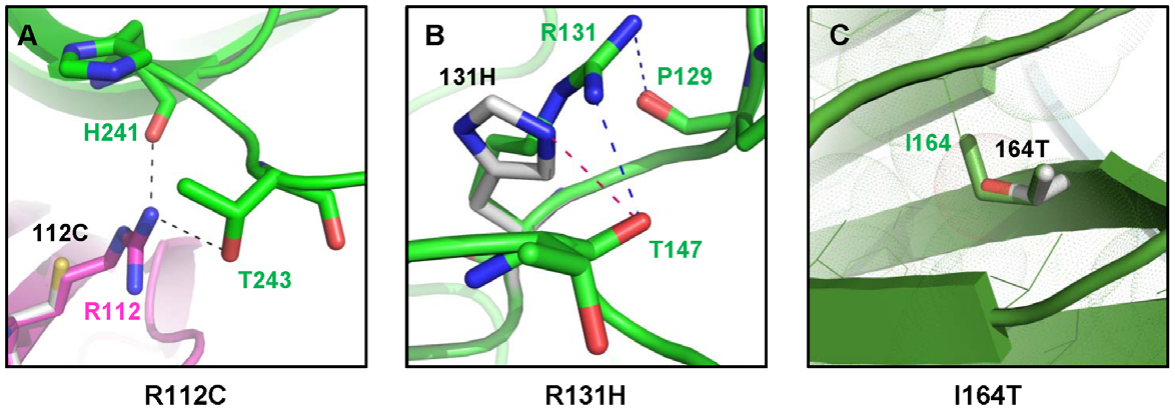
Local structural alterations of amino acid substitutions (R112C, R131H, and I164T) in modeled human adiponectin globular domain. Wild-type and variant adiponectin trimer are superimposed. Three monomers of adiponectin wild-type are indicated in green, cyan, and magenta and labeled with the color letters corresponding to their chain. The variant residues are showed with the gray sticks and labeled with the black letters. The side chains of amino acids within hydrophobic core are shown in line structure with surrounding dots. The O, N, and S atoms are shown with red, blue, and yellow, respectively. A–C: The local structural alterations for R112C, R131H, and I164T variants, respectively.

The G84R and G90S variants are located in the collagenous domain (Figure 2A). To date, the crystal structure of adiponectin collageneous domain is unavailable. Therefore, the 3D structure of these two variants could not be analyzed. However, they reside in Gly-X-Y repeats, the highly conserved region which is important for triple helix formation and forming of higher degree of adiponectin oligomers. Substitutions of glycine with either arginine (G84R) or serine (G90S) cause the decrease in Gly-X-Y repeated numbers and impaired adiponectin multimerization [15]. Only the crystal structure of mouse adiponectin globular domain is available [16]. We therefore created a structure of human adiponectin globular domain (Figure 3) by homology modeling using a 30 kD adipocyte complement-related protein precursor – ACRP30 (PDB 1C28), the most suitable template identified by blast searches, as a template. The alignments of mouse template (residues 114–245) and human (residues 111–242) globular domain sequences showed 97.91% of sequence identity without any gaps. The superimpositions of template and all human adiponectin variants showed the root mean square (RMS) values of 0.89 Å, 0.67 Å, and 0.57 Å for chain A, B, and C, respectively. It was found that R112C and I164T, which did not form any adiponectin oligomeric classes and exhibited secretion defects (Table 1), showed the alteration of the trimer interface. R112C was likely to cause loss of H-bonds between monomers (Figure 4A). The introduction of the hydroxyl group of threonine (T) instead of the hydrophobic side chain of isoleucine (I) at residue 164 was likely to disturb the hyprophobic core within adiponectin monomers (Figure 4C). The correlation between the *in silico* analysis performed in this study, and the results from previous *in vitro* experiments, suggests the potentiality in the use of amino acid alignment together with *in silico* mutagenesis for prediction of the effect of amino acid substitutions on adiponectin oligomerization.

In the present study, three novel adiponectin variants including R55H, R112H, and R131H were identified (Figure 2A). Arginine at residues 55 (Figure 2B) and 131 (Figure 2C) are conserved among all available species and the R55H and R131H variants were identified only in T2D patients but not non-diabetic controls. R55 resides in the highly conserved Gly-X-Y motif in the collagenous domain; thus, it was speculated to affect the formation of adiponectin HMW multimers, the same as was observed for the G84R and G90S variants. *In silico* modeling of R131H revealed the alteration of H-bond forming patterns within the globular domain (Figure 4B). Therefore, we hypothesized that R131H might impair adiponectin oligomerization and secretion, the same as that demonstrated for R112C and I164T. R112H was identified in a non-diabetic individual but not T2D patients. In addition, amino acid residue 112 in the sequence alignment can be either arginine (R) or histidine (H) (Figure 2C). Therefore, R112H was speculated to exhibit normal protein oligomerization.

### Expression, secretion, and oligomerization of novel adiponectin variants

**Figure 5:**
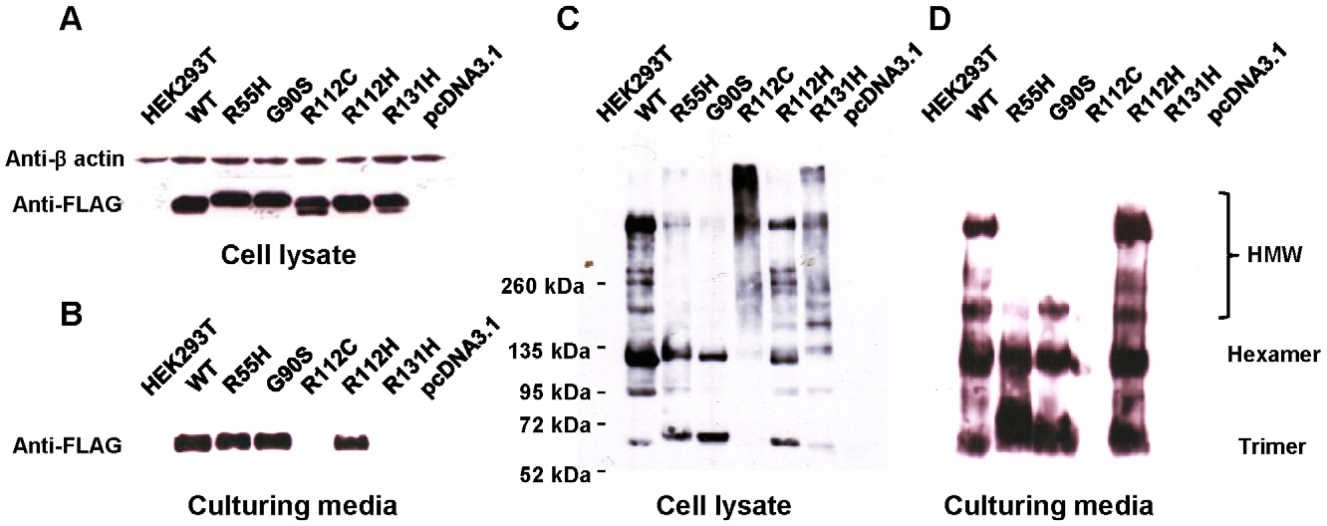
Expression, secretion, and oligomerization of adiponectin variants. Cell lysate (A) and culturing media (B) of HEK293T cells transiently expressed adiponectin wild-type and variants under reducing and heat-denaturing condition. Cell lysate (C) and culturing media (D) of HEK293T cells transiently expressed adiponectin wild-type and variants under non-reducing and non heat-denaturing condition. The total protein of each sample was equally loaded. Results shown are typical of three independent experiments.

In order to investigate the impact of the three novel adiponectin variants (R55H, R112H, and R131H) on protein oligomerization and secretion, the recombinant proteins of these three novel and the other two reported variants, G90S and R112C, which exhibited multimerization and secretion defects [15], respectively, were transiently expressed in cultured HEK293T cells. The expression, secretion, and oligomerization of these variants were investigated. All variants could be expressed (Figure 5A); however, R112C and R131H variants were undetectable in culturing media (Figure 5B). Under non-reducing and non heat-denaturing conditions, adiponectin wild-type and R112H variant clearly exhibited three oligomeric forms (Figure 5C). In contrast, small amounts of HMW of R55H and G90S variants were detected both in cell lysate (Figure 5C) and in culturing media (Figure 5D). In cell lysate, R112C variant exhibited protein aggregation while R131H variant showed combined patterns of protein aggregation and abnormal oligomerization (Figure 5C), which were correlated with secretion defect.

Since all variants identified in the studied subjects were in heterozygous conditions, co-expression of wild-type and adiponectin variants was carried out to determine whether the variant proteins have any effect on the wild-type protein or not. The cDNA sequence encoding wild-type protein was tagged with either c-Myc or FLAG sequence while all of those encoding variant proteins (R55H, G90S, R112C and R131H) were tagged with FLAG sequence.

**Figure 6:**
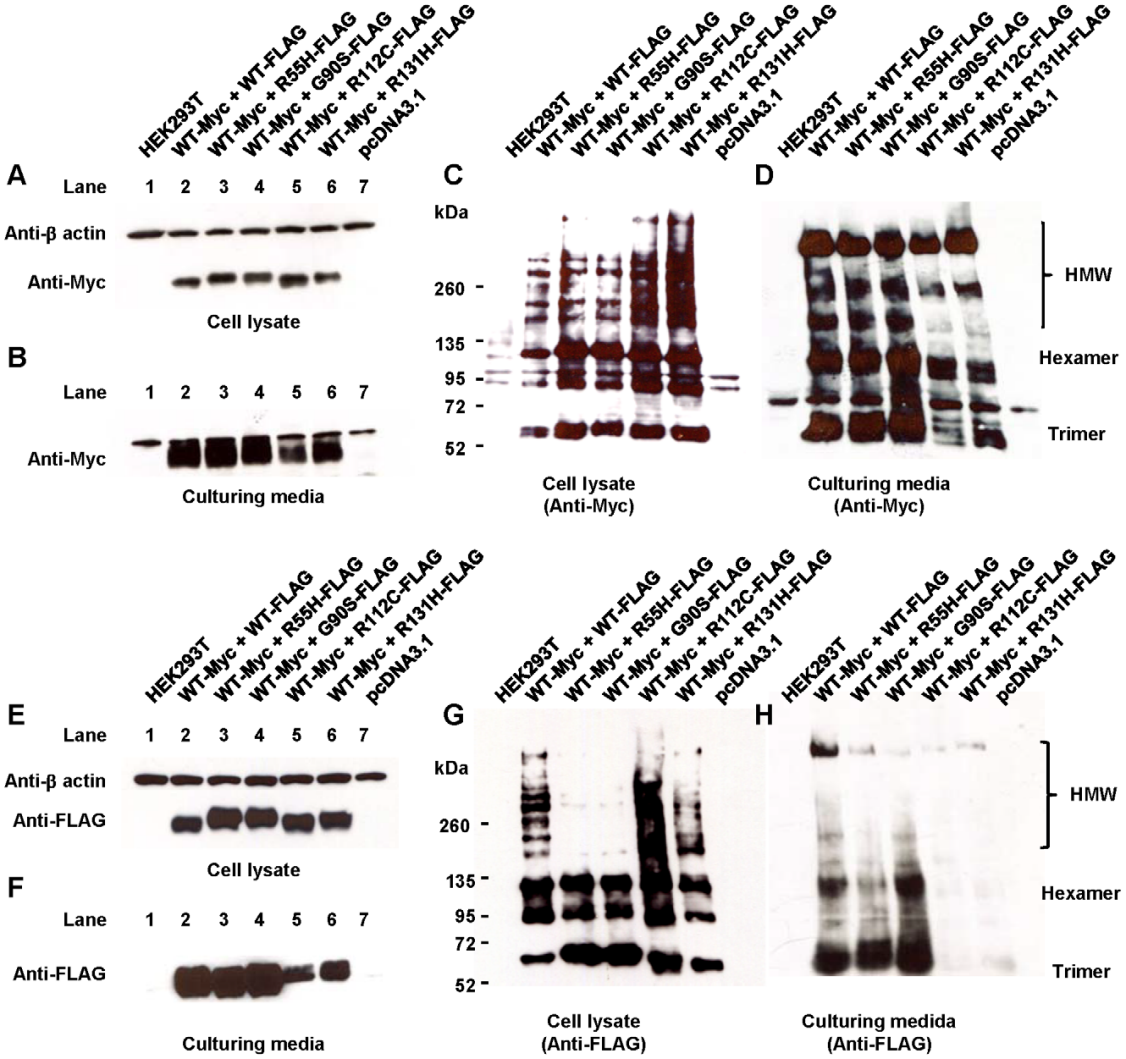
Co-expression of wild-type and variant adiponectins. Adiponectin variants tagged with FLAG sequence were co-expressed with wild-type tagged with c-Myc sequence. A–D: Immunoblotting of wild-type protein by using anti-Myc antibody. E–H: Immunoblotting of variant proteins by using anti-FLAG antibody. A, B, E and F: Cell lysates (A and E) and culturing media (B and F) of adiponectin variants co-expressed with wild-type under reducing and heat-denaturing condition. C, D, G, and H: Cell lysate (C and G) and culturing media (D and H) of adiponectin variants co-expressed with wild-type under non-reducing and non heat-denaturing condition. The total protein of each sample was equally loaded. Results shown are typical of three independent experiments.

Under the conventional SDS-PAGE, both cell lysate and culturing media of HEK293T cells expressing wild-type protein alone and those co-expressing wild-type proteins and either R55H or G90S variant exhibited the similar protein levels when they were detected with either anti-Myc (Figures 6A and 6B) or anti-FLAG (Figures 6E and 6F) antibody. Under non-reducing and non-heat denaturing conditions, HMW multimers of the wild-type protein co-expressed with either R55H or G90S could be detected at the same levels as the wild-type protein when it was expressed alone (Figures 6C and 6D). However, HMW multimers of the variant proteins were barely detected (Figures 6G and 6H). These results suggested that the formation of wild-type HMW multimers was not interfered by the R55H and G90S variants which are defective in multimerization.

In cell lysate, the level of wild-type protein expressed alone and wild-type proteins co-expressed with either R112C or R131H variant were similarly detected by conventional SDS-PAGE (Figure 6A). The levels of R112C and R131H variants were similar to that of the wild-type protein when they were detected by anti-FLAG antibody (Figure 6E). However, in culturing media, the wild-type proteins when co-expressed with either the R112C or R131H variant was present at lower levels as it was compared to that of the wild-type protein expressed alone (Figure 6B), suggesting that the two variant proteins could induce the wild-type protein to present secretion defects. The levels of R112C and R131H variants were slightly present in culturing media (Figure 6F), suggesting that the lower amounts of variant proteins could be secreted when they were co-expressed with wild-type protein. Under non-reducing and non-heat denaturing conditions, wild-type protein co-expressed with either R112C or R131H in cell lysate were found to be aggregated (Figure 6C) and lower amounts of their HMW multimers were observed in culturing media, as compared to those of wild-type expressed alone (Figure 6D). Thus, the R112C and R131H variants could exert a dominant negative effect over the wild-type protein, causing the secretion defect of the wild-type protein by sequestering into their co-aggregated forms. All oligomeric forms (and some aggregated forms) of R112C and R131H variants were detected in cell lysate (Figure 6G), suggesting that some hetero-oligomers between the wild-type and variant proteins could be formed. In addition, low levels of these hetero-oligomers could be secreted in culturing media (Figures 6F and 6H).

### Sub-cellular localization of wild type and variant adiponectins

**Figure 7:**
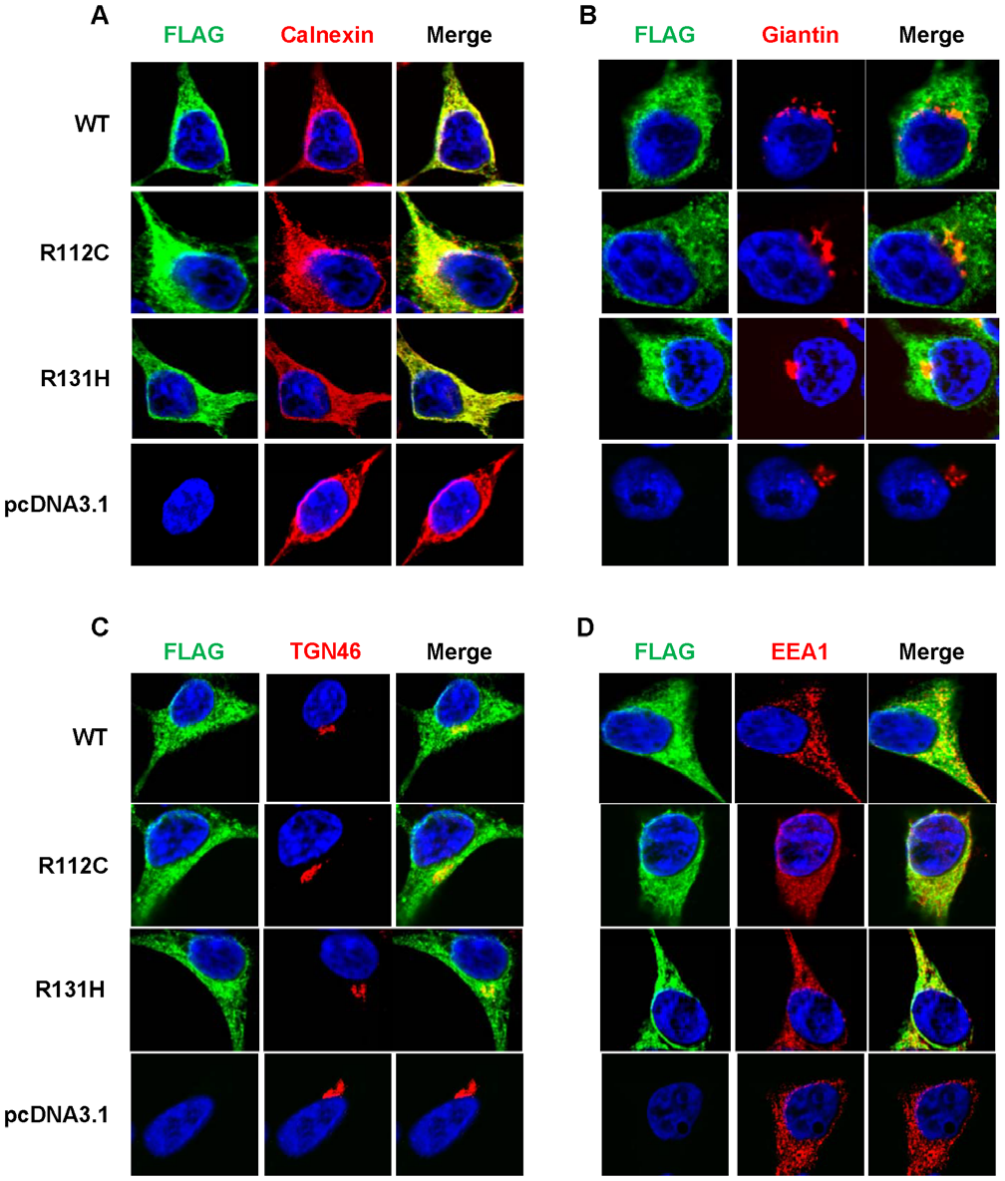
Sub-cellular localizations of wild-type, R112C and R131H adiponectins. The wild-type, R112C and R131H adiponectins linked with FLAG epitope transiently expressed in HEK293T cells were detected with mouse anti-FLAG antibody, and with either rabbit anti-Calnexcin (A), rabbit anti-Giantin (B), rabbit anti-TGN46 (C) or rabbit anti-EEA1 (D) antibody, which are antibodies specific to ER, Golgi, TGN and early endosome markers, respectively. Goat anti-mouse IgG-conjugated with Alexa Fluor® 488 (green) and donkey anti-rabbit IgG-conjugated with Cy3 (red) were secondary antibodies for staining adiponectin and the organelle markers, respectively. The nucleus was stained with Hoechst 33342 fluorescent dye (blue).

It has been reported that a substantial amount of adiponectin in the steady state of 3T3-L1 adipocytes was located in the Golgi/TGN [17] and it was released via the endosomal compartment [18]. To examine the intracellular trafficking underlying the secretion defects of adiponectin variants, HEK293T cells were transfected with the plasmid constructs containing either wild-type or variant (R112C and R131H) cDNA tagged with the FLAG sequence to observe their expression and localization with the intracellular organelle markers. These included Calnexin, Giantin, TGN46, and an early endosome antigen 1 (EEA1) which are the organelle markers for endoplasmic reticulum (ER), Golgi apparatus, trans-Golgi network and early endosome, respectively. The results showed that wild-type and variant (R112C and R131H) adiponectins were similarly co-localized with all organelle markers studied (Figure 7). The localizing coefficients and co-localizing intensities of the two variants and all organelles were not different from those of the wild type proteins (data not shown).

### Intracellular degradation of adiponectin

**Figure 8:**
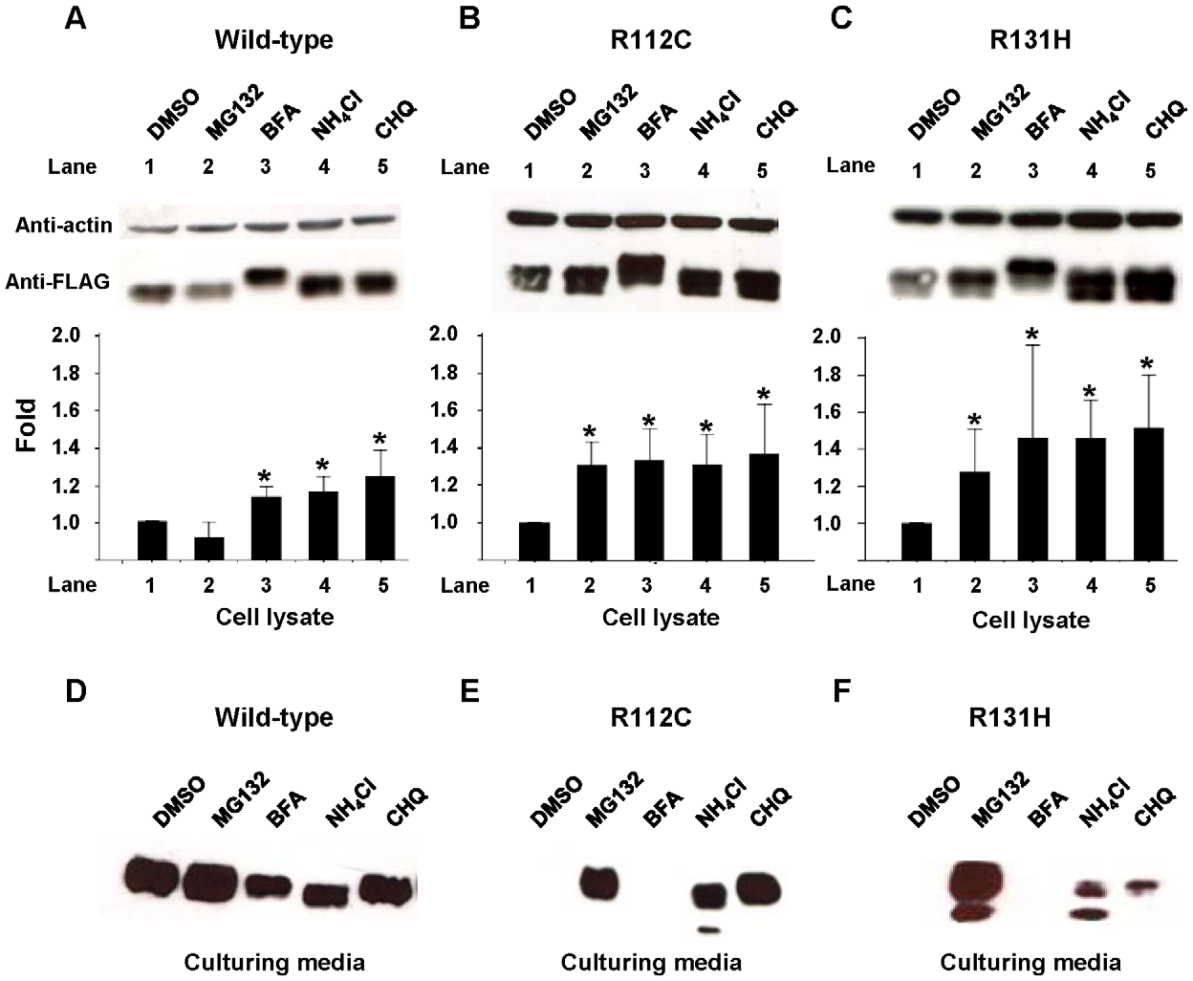
Investigation of lysosomal degradation of wild-type, R112C and R131H adiponectins. At 24 hours after transfection, HEK293T cells were treated with proteasomal/lysosomal or lysosomal inhibitors for 8 hours. The concentrations of reagents are as followed: MG132 10 mM, BFA 5 µg/mL, NH_4_Cl 25 mM and chloroquine (CHQ) 100 µM. Equal amount of protein from each sample was immunoblotted with anti-FLAG. A–C: Intracellular adiponectin levels of wild-type (A), R112C (B) and R131H (C) after treating with degradation inhibitors. The bar graphs represented relative levels of adiponectin (adiponectin/actin) represented in folds of samples treated with DMSO. Data are mean ± SD of 3 independent experiments. * *P* value<0.05, analyzed by Mann-Whitney U test. D–F: Extracellular adiponectin levels of wild-type (D), R112C (E) and R131H (F) after treating with degradation inhibitors. Results shown are typical of 3 independent experiments.

Generally, misfolded and unassembled proteins in ER are transported to be degraded via the proteasomal system in cytoplasm. However, the non-secreted R112C and R131H variants could traffic from ER through Golgi, trans-Golgi network, and early endosome (Figures 7A–7D). Thus, we hypothesized that the lysosomal system should be the site for their degradations. To test our hypothesis, the levels of intracellular and secretory forms of wild-type protein, R112C and R131H variants in the presence of proteasomal/lysosomal and lysosomal inhibitors were examined. In the presence of MG132 – a proteasome inhibitor which also inhibits lysosomal cysteine proteases, the intracellular level of wild-type protein was slightly but not significantly decreased (Figure 8A). This was correlated with the finding that its secretion was promoted by this inhibitor (Figure 8D). Brefeldin A (BFA) – an antibiotic that inhibits transport of proteins from ER to Golgi and induces retrograde protein transport from the Golgi to the ER, significantly increased the intracellular level of wild-type protein by abatement of its secretion (Figures 8A and 8D). Inhibition of lysosomal hydrolase activity by NH_4_Cl and chloroquine significantly increased the intracellular level of wild-type protein (Figure 8A) by diminishing its secretion (Figure 8D) rather than reducing its degradation. In contrast, MG132 increased both intracellular and extracellular levels of R112C (Figures 8B and 8E) and R131H variants (Figures 8C and 8F), suggesting that inhibition of proteasomal and lysosomal activities by MG132 could decrease their degradation while promoting their secretion. After holding proteins within ER by BFA, the intracellular levels of these two variants were significantly increased. These results suggested that chemical retention of variant proteins, within the ER, rescued proteins from degradation. Thereby, post-ER organelles are likely responsible for their degradation. The finding that NH_4_Cl and chloroquine significantly increased intracellular levels of R112C and R131H variants confirmed the role of lysosome in degradation of these two variants. Additionally, inhibitions of lysosomal degradation by NH_4_Cl and chloroquine could also promote secretion of the two adiponectin variants (Figures 8E and 8F). Taken together, these results suggested the role of the lysosomal compartment in the turnover of R112C and R131H variants, but not the wild-type protein.

## Discussion

Herein, we reported novel adiponectin variants identified in Thai patients with T2D and demonstrated the effects of amino acid substitutions on structural alterations and the defects in their biochemical properties. The novel R55H and R131H variants were likely to be implicated in T2D pathogenesis by affecting adiponectin multimerization and secretion, respectively. Since the patients who carried these two variants had taken anti-hyperglycemic drugs that affect plasma adiponectin levels, the plasma adiponectin levels in these patients were not measured. The decreased adiponectin levels in these patients were suspected as implied from the observation that the novel R55H and R131H variants exhibited the same characteristics as the reported G90S and R112C variants, respectively, which were found to be associated with hypoadiponectinemia [15] (Table 1). It should be noted that the patients who carried these two novel variants exhibited greater waist circumferences than the average value of the patients with T2D (Table 2), pointing out the clinical characteristics that associate with adiponectin abnormalities. These variants were found among T2D patients, but not in a sample of non-diabetic subjects. Collapsing these two functional variants together for analysis did not show significant association with T2D (p>0.05, data not shown). Additionally, an association between these variants and T2D cannot be determined with certainty because of the low power associated with the small sample size and the low frequencies of these variants.

**Table 2:** Clinical and laboratory characteristics of studies subjects.

								T2D carrying non-synonymous variant		
Clinical and laboratory parameter	T2D			Non-diabetic control			*p* value	R55H*	R131H	H241P
Number (male/female)	202 (58/144)			210 (58/147)			-	2 (0/2)	1 (1/0)	1 (0/1)
Age (year)	53.75	±	11	53.34	±	9.06	NS	65	55	59
Age at diagnosis (year)	49.39	±	10.87		-		-	65	53	53
Weight (kg)	68.58	±	12.47	59.08	±	9.83	<0.001	70	84.7	69
BMI (kg/m^2^)	27.84	±	4.95	23.85	±	3.2	<0.001	27.24	27.66	27.64
Waist (cm)	87.99	±	10.9	82.24	±	9.29	<0.001	92	100	86
Hip (cm)	99.82	±	9.73	95.68	±	6.69	<0.001	110	105	94
Waist/hip ratio	0.89	±	0.07	0.86	±	0.06	<0.001	0.84	0.95	0.91
Systolic BP (mm Hg)	132.14	±	16.16	115.5	±	14.04	<0.001	120	140	200
Diastolic BP (mm Hg)	81.43	±	10.4	70.94	±	9.35	<0.001	70	74	110
FPG (mmol/L)	11.33	±	5.1	4.88	±	0.35	<0.001	18.93	5.77	10.93
HbA1c (%)	8.29	±	2.49		-		-	12	7.4	10.1
Total cholesterol (mmol/L)	5.86	±	1.2	5.46	±	0.94	<0.001	5.96	3.08	5.80
Triglyceride (mmol/L)	2.02	±	1.02	1.17	±	0.49	<0.001	1.41	1.21	2.16
LDL-C (mmol/L)	3.71	±	1.08	3.23	±	0.86	<0.001	4.43	1.18	3.68
HDL (mmol/L)	1.27	±	0.31	1.59	±	0.42	<0.001	1.06	1.35	1.14

Data are mean ±SD. The *p* values compare clinical and laboratory data between 202 of T2D patients whose data are completely available and 210 non-diabetic controls by using either Mann–Whitney U or student's T-test.

*Data of one from two patients carrying R55H.

The adiponectin collagenous domain is crucial for stabilizing of adiponectin trimers and further formations of hexamers and HMW multimers via interdisulfide bonds [19]. The R55H, G84R, and G90S variations did not disturb adiponectin trimeric and hexameric formations but obstructed their multimerization. These variants are not close to interdisulfide bond forming site (Cys^36^) and they were still capable of forming hexamers. However, they might cause conformational change and conceal the remaining free thiol from interacting with other hexamers. Another possible mechanism responsible for multimerization defect might be due to ineffectiveness of these variants in interacting with ER chaperones, such as ERp44 [20] or Ero1-α [21], which assist the formation of adiponectin multimers. It is remarkable that molecular sizes of the R55H and G90S variants were higher than that of the wild-type, implying a gain-of-glycosylation which has been reported as a characteristic of rare mutations implicated in several diseases [22]. However, whether R55H and G90S are gain-of-glycosylation variants, and exactly what is the precise mechanism responsible for their multimerization defects, are still to be elucidated.

Adiponectin globular domain is crucial for initiation of trimer formation through hydrophobic interaction. Variations occurred at the globular domain may impair adiponectin trimerization affecting protein secretion. These included the previously reported R112C and I164T [15] as well as the novel R131H variants. We found that protein aggregation exhibited by the R112C and R131H variants disappeared in the presence of a reducing agent (data not shown). These findings suggested disulfide-linked aggregation of these two variants. Generally, protein folding is determined by hydrophobic interactions and hydrogen bonding. The breaking of H-bonds between R112C monomers and the alteration of H-bond forming patterns within R131H monomers, as suggested by *in silico* modeling and mutagenesis, may obstruct the formation of adiponectin trimers. The resulting free thiol may cause incorrect disulfide cross-linking that participates in protein aggregation [23].

While the R55H and G90S variants did not interfere with wild-type protein in the forming of HMW multimers, we showed that the R112C and R131H variants could form aggregated complexes with the wild-type protein, resulting in decreasing wild-type protein secretion. However, some forms of hetero-oligomers between the wild-type and mutant proteins could be secreted from the cultured HEK293T cells. These secreted hetero-oligomers might be predominated with the wild-type protein.

It is known that proteasomal and lysosomal systems are the two major pathways for intracellular degradation of the misfolded and unassembled proteins. Abnormal proteins, which are destined to proteasomal degradation, are usually retained within ER before being degraded by the proteasome pathway. However, the R112C and R131H variants were not found to be accumulated in ER; they trafficked to Golgi, TGN, and even endosomes but could not be secreted. Therefore, we hypothesized that the lysosomal system should play a role in the degradation of these two variants. The increases of both intracellular and extracellular levels of these two variants in the presence of lysosomal inhibitors supported our hypothesis. Nonetheless, the increase in intracellular levels of wild-type proteins by lysosomal inhibitors was probably attributable to a decrease in its secretion, rather than prevention from its degradation. Consistent with this study, Blumer *et al.* have demonstrated that the acidotropic agents inhibited secretion of endogenous adiponectin from adipocytes by interrupting an acidic interior of vesicular pathway and showed that the lysosomal system was not responsible for degradation of endogenous (or wild-type) adiponectin [24]. It is possible that misfolded or unassembled variants, but not wild-type adiponectin, are degraded by the lysosomal system, and inactivation of lysosomal compartments allows these abnormal proteins to escape from the degradation system to be secreted into the culturing media.

We have shown that inhibition of protein degradation by MG132 could promote secretion of the R112C, R131H, and wild-type adiponectins. In the same way, MG-132 treatment of mdx mice rescued the expression and membrane localization of dystrophin, the protein which is absent in the skeletal muscle of Duchenne muscular dystrophy (DMD) patients and mdx mice [25]. This finding is also similar to the increased secretion of the mutant Z form of α1-antitrypsin (α1-AT) which is responsible for greater than 95% of individuals with α1-AT deficiency syndrome by inhibition of intracellular degradation using proteasomal inhibitors [26]. In addition, proteasome inhibitions also increased the secretion of active BACE, an aspartic protease involved in the production of toxic peptide accumulation in the brain of Alzheimer patients [27]. In most of the cases, it is likely that inhibition of proteasome activities decreases the opportunity of misfolded proteins retained in ER to be degraded, thereby prolonging their time in the ER lumen, and increasing the chance to be retrieved by vesicular trafficking. However, the R112C and R131H variants can traffic from ER to Golgi, trans-Golgi network, and early endosome but can not be secreted, and the lysosomal, rather than proteasomal, system is responsible for their degradations. Thus, MG132 may rescue their secretion by other mechanisms. One possibility is that MG132 elevates a number of ER chaperones necessary for protein folding [28]. There is evidence indicating that misfolded proteins can be refolded successfully by modifying the molecular chaperone environment using proteasome inhibitors, including MG132 [29], [30]. For example, enzymatic function of a cystathionine β-synthase (CBS) variant, T262M, could be rescued by MG132 and was not associated with increased levels of CBS T262M variant. It has also been shown that the rescuing effect of MG132 was accompanied by elevation of Hsp70 chaperone content but was not due to inhibition of mutant protein degradation [30]. Thus, it is likely that MG132 may promote secretion of the R112C and R131H variants through its effect on molecular chaperones in ER.

In summary, we have identified novel candidate variants for T2D in the *ADIPOQ* gene in the Thai population. R55H variant impairs protein mutimerization while the novel R131H variant causes secretion defect in a dominant negative fashion. We have also demonstrated that secretion defect is unlikely to result from retention of the variant proteins in ER and the lysosomal compartment plays a role in degradation of these variants. We have also illustrated that MG132 can enhance wild-type protein secretion and rescue adiponectin variants from secretion defect. This suggests the benefit of using protease inhibitors or other compounds that modify the chaperone environment in ER as therapeutic agents for the treatment of patients carrying adiponectin variants with secretion defect.

## Materials and Methods

### Subjects and assessment of clinical data

A total of 272 of T2D subjects were recruited at Siriraj Hospital Diabetic Clinic. The criteria for diagnosis of diabetes mellitus followed that were described by the American Diabetes Association (ADA) 1997. With this sample size, we had 93.4% probability of detecting mutations with a frequency in the T2D population as low as 0.5% and 99.6% probability to detect mutations with a frequency as low as 1%. Ethical approval for the study was granted by the Ethics Committee of the Faculty of Medicine Siriraj Hospital. All subjects were informed for the purpose and extent of the study before signing consent forms and enrollment into the study. The patients' plasma glucose was determined by glucose oxidase method. Glycosylated hemoglobin (HbA_1C_), total cholesterol, triglycerides, and HDL-cholesterol were assayed by standard procedures. LDL-cholesterol was calculated by Friedewald formula or measured directly when appropriate. A number of 210 non-diabetic controls were volunteers recruited at Siriraj Hospital Check-up Center, Department of Preventive and Social Medicine, Faculty of Medicine Siriraj Hospital, Mahidol University. The selection criteria included healthy individuals with (*i*) age more than 40 years, (*ii*) fasting plasma glucose levels less than 100 mg/dl, and (*iii*) no family history of diabetes in their first degree relatives.

### Analyses of *ADIPOQ* gene

DNA samples were extracted from EDTA anti-coagulated venous blood samples by a standard phenol/chloroform method. Regulatory region (11.5–10.33 kb upstream of the start codon), 5′ untranslated region, exon-intron boundaries, and coding regions of *ADIPOQ* were analyzed for sequence variations by polymerase chain reaction and single strand conformation polymorphism (PCR-SSCP) method, followed by direct sequencing. The details of primers and optimal PCR-SSCP conditions are provided in Table S2.

### *In silico* investigation of rare non-synonymous variants

The three dimensional structure of both wild-type and variant human adiponectin were constructed by homology modeling using the SWISS-MODEL workspace [31]. The modeling steps included: (*i*) identification of the most suitable template, (*ii*) alignment of target sequence and template structure, (*iii*) model building, and (*iv*) model quality evaluation. Alterations of H-bond forming pattern caused by variant proteins were examined on PyMOL (DeLano Scientific LLC, CA, USA).

### Plasmid construction, transfection, and preparation of samples for analysis

*ADIPOQ* coding region (NM_004797.2) with either the FLAG (DYKDDDDK) or c-Myc (EQKLISEEDL) tagged sequences at the carboxyl terminus was generated from a human kidney cDNA library using the primers exhibited in Table S3 and cloned into pcDNA3.1(+), between *BamH*I and *EcoR*I sites. Plasmid constructs containing each of three novel variants (R55H, R112H, and R131H) and two previously reported ones (G90S and R112C) [15] were generated by PCR site-directed mutagenesis using the wild-type adiponectin tagged with FLAG as a template. The plasmid constructs were transfected into HEK293T cells cultured in DMEM/F-12 supplemented with 10% FBS by using Lipofectamine 2000 (Invitrogen, CA, USA). Cells were harvested at 48 hours after transfection. Cell lysate was prepared using RIPA buffer (1% NP-40, 0.5% deoxycholate, 0.1% SDS, 150 mM NaCl, 20 mM Tris-HCl pH 7.4, 5 mM EDTA pH 8.0), supplemented with protease inhibitor cocktail (Roach Diagnotics GmbH, Mannheim, DE). The culturing media was cleared by centrifugation at 13,500 rpm for 1 hour, and dialyzed against distilled water for 24 hours before concentrating by lyophilization. The lyophilized media was dissolved with a low salt buffer (20 mM HEPES, 1 mM CaCl_2_). Total protein contents in prepared samples were quantified by Bradford protein assay.

### SDS-PAGE and Western blot analysis

Cell lysate and culturing media were prepared for SDS-PAGE as described by Waki *et al* [15]. For investigation of protein expression and secretion, samples were heated in the presence of β-mercaptoethanol before being separated by 12% SDS-PAGE. Their multimerizations were investigated by using 2–12% gradient SDS-PAGE under non-reducing and non-heat denaturing condition [15]. Proteins were transferred to the nitrocellulose membrane and then blocked with 3% skim milk in TBS-T before incubating with either anti-FLAG® M2 Monoclonal antibody (Sigma, MO, USA), c-Myc antibody (Santa Cruz Biotechnology, CA, USA), or anti-actin antibody (Santa Cruz Biotechnology, CA, USA) diluted in TBS-T containing 3% skim milk. After washing, the membrane was incubated with the HRP-conjugated secondary antibody (Dako, Glostrup, Denmark), and visualized with the enhanced chemiluminescence (ECL) system (Promega, WI, USA). Relative quantities of the proteins were determined by using the ImageJ program [32].

### Immunofluorescent staining and co-localization analysis

HEK293T cells were grown on glass coverslips overnight, before transfection with plasmid constructs containing wild-type or variant (R112C or R131H) adiponectin cDNA tagged with FLAG sequence. At 24 hours after transfection, cells were fixed in 2% paraformaldehyde in PBS, permeabilized by 0.2% Tritron-X in PBS, washed with 1% FBS in PBS, and immunostained with mouse anti-FLAG® M2 Monoclonal antibody for adiponectin and either rabbit anti-Calnexin (Santa Cruz Biotechnology, CA, USA), rabbit anti-Giantin (Abcam, Cambridge, UK), rabbit anti-TGN46 (Abcam, Cambridge, UK) or rabbit anti-EEA1 (Abcam, Cambridge, UK) antibodies diluted in 1% FBS in PBS for 1 hour. After discarding the primary antibodies, cells were washed with 1% FBS in PBS before incubating with secondary antibodies conjugated with fluorescent dye for another 1 hour. Each secondary antibody mixture contained Alexa Fluor® 488-conjugated goat anti-mouse (Molecular Probes, Eugene, USA), Cy3-conjugated donkey anti-rabbit IgG (Jackson Immunoresearch Laboratories, West Grove, USA), and Hoechst 33342 dye (Invitrogen, Carlsbad, USA) for the staining of recombinant adiponectin, organelle markers, and nuclei, respectively. The coverslips were washed with 1% FBS in PBS and then mounted with 50% antifade reagent. The immunofluorescent images were examined and captured by confocal laser scanning microscope (Zeiss LSM 510 META).

## Supporting Information

Figure S1 **Mobility shift pattern from SSCP analysis and nucleotide sequence analysis of novel variations identified in the present study.**
(DOC)

Table S1 ***ADIPOQ*** **variants identified in the present study.**
(DOC)

Table S2 **Primers and PCR-SSCP conditions for screening of** ***ADIPOQ*** **variations.**
(DOC)

Table S3 **Primers for amplification of** ***ADIPOQ*** **cDNA tagged with either FLAG or c-Myc sequences.**
(DOC)
