# Supplementary material for: Novel Adiponectin Variants Identified in Type 2 Diabetic Patients Reveal Multimerization and Secretion Defects
Source: PLoS One. 2011 Oct 26;6(10):e26792. doi: 10.1371/journal.pone.0026792 (PMC3202584; doi:10.1371/journal.pone.0026792)
Supplement: Figure S1 — Mobility shift pattern from SSCP analysis and nucleotide sequence analysis of novel variations identified in the present study. (DOC) [file pone.0026792.s001.doc]

**Figure S1**


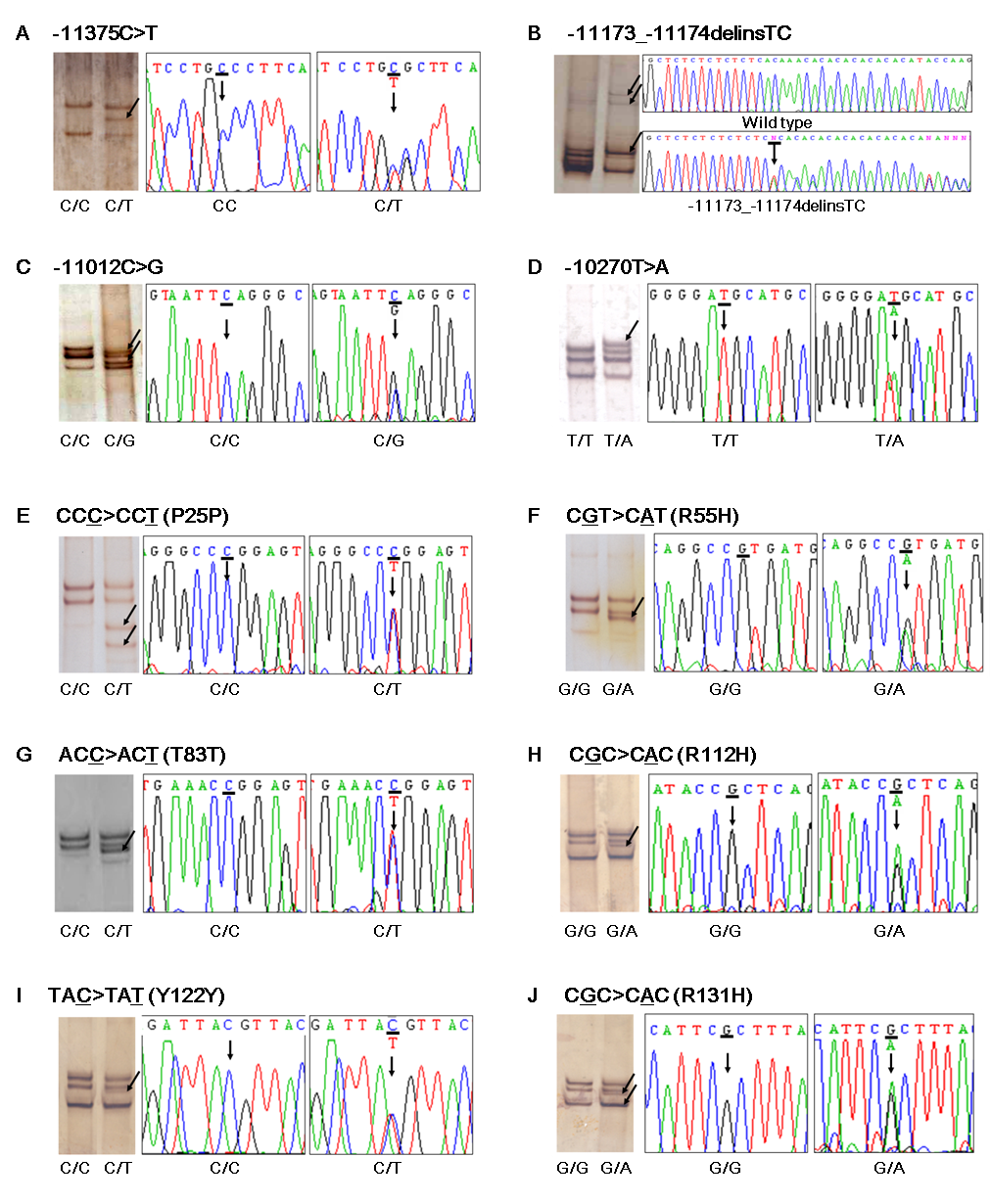
**Mobility shift pattern from SSCP analysis and nucleotide sequence analysis of**

**novel variations identified in the present study**
