# Supplementary material for: Novel Adiponectin Variants Identified in Type 2 Diabetic Patients Reveal Multimerization and Secretion Defects
Source: PLoS One. 2011 Oct 26;6(10):e26792. doi: 10.1371/journal.pone.0026792 (PMC3202584; doi:10.1371/journal.pone.0026792)
Supplement: Table S1 — ADIPOQ variants identified in the present study. (DOC) [file pone.0026792.s002.doc]

**Table S1 *ADIPOQ* variants** identified in the present study

| **No.** | **Fragment** | **Region** | **Nucleotide change** | **Codon change** | **Amino acid change** | **SNP accession** |
| --- | --- | --- | --- | --- | --- | --- |
| 1 | Promoter 1 | Promoter | -11423A>G | - | - | rs16861194 |
| 2 | Promoter 1 | Promoter | -11388G>A | - | - | rs17300539 |
| 3 | Promoter 1 | Promoter | -11375C>T | - | - | Novel |
| 4 | Promoter 1 | Promoter | -11374C>G | - | - | rs266729 |
| 5 | Promoter 2 | Promoter | -11173_-11174delinsTC | - | - | Novel |
| 6 | Promoter 2 | Promoter | -11154_-11155delinsCA | - | - | rs60806105 |
| 7 | Promoter 3 | Promoter | -11012C>G | - | - | Novel |
| 8 | Promoter 7 | Promoter | -10270T>A | - | - | Novel |
| 9 | Exon 2-1 | Exon 2 | 45T>G | GGT>GGG | G15G | rs2241766 |
| 10 | Exon 2-1 | Exon 2 | 75C>T | CCC>CCT | P25P | Novel |
| 11 | Exon 2-1 | Exon 2 | 164G>A | CGT>CAT | R55H | Novel |
| 12 | Exon 2-2 | Intron 2 | 276G>T | - | - | rs1501299 |
| 13 | Exon 3-1 | Exon 3 | 1160C>T | ACT>ATT | T83T | Novel |
| 14 | Exon 3-2 | Exon 3 | 1246G>A | CGC>CAC | R112H | Novel |
| 15 | Exon 3-2 | Exon 3 | 1277C>T | TAC>TAT | Y112Y | Novel |
| 16 | Exon 3-2 | Exon 3 | 1303G>A | CGC>CAC | R131H | Novel |
| 17 | Exon 3-3 | Exon 3 | 1572C>A | CGT>AGT | R221S |  |
| 18 | Exon 3-3 | Exon 3 | 1633A>C | CAC>CCT | H241P |  |

1. Kondo H, Shimomura I, Matsukawa Y, Kumada M, Takahashi M, et al. (2002) Association of adiponectin mutation with type 2 diabetes: a candidate gene for the insulin resistance syndrome. Diabetes 51: 2325-2328.

2. Hara K, Boutin P, Mori Y, Tobe K, Dina C, et al. (2002) Genetic variation in the gene encoding adiponectin is associated with an increased risk of type 2 diabetes in the Japanese population. Diabetes 51: 536-540.
