# Supplementary material for: Novel Adiponectin Variants Identified in Type 2 Diabetic Patients Reveal Multimerization and Secretion Defects
Source: PLoS One. 2011 Oct 26;6(10):e26792. doi: 10.1371/journal.pone.0026792 (PMC3202584; doi:10.1371/journal.pone.0026792)
Supplement: Table S2 — Primers and PCR-SSCP conditions for screening of ADIPOQ variations. (DOC) [file pone.0026792.s003.doc]

**Table S2 Primers and PCR-SSCP conditions for screening of *ADIPOQ* variations**

| **Amplicon** | **Nucleotide sequence (5’🡪3’)** | **Product Size** | **Annealing temperature (°C)** | **SSCP condition** |
| --- | --- | --- | --- | --- |
| **Promoter 1** | **F**-ACTTGCCCTGCCTCTGTCTG | 250 | 60 | 10% polyactylamide |
|  | **R**-GCCTGGAGAACTGGAAGCTG |  | With 10%DMSO | 10% Glycerol |
|  |  |  |  | 100 volt 5.30 hrs. |
| **Promoter 2** | **F**-CCAAGAAAGTCCAAGGTG | 386 | 58 | 10% polyacryamide |
|  | **R**-AAGTTCCTGGAGTGGTGT |  |  | 0% Glycerol |
|  |  |  |  | 120 volt 3.30 hrs. |
| **Promoter 3** | **F**-GCTGTCAAAATGTTATCCGA | 291 | 55 | 10% polyacryamide |
|  | **R**-TCAGAGGGGTCTGCAATC |  |  | 5% Glycerol |
|  |  |  |  | 20 mA 4.30 hrs |
| **Promoter4** | **F**-GGTCAGAGAGTGGAGGATGTG | 208 | 55 | 10% polyacryamide |
|  | **R**-TAATTGCTCAGTGGTCCAGG |  |  | 5% Glycerol |
|  |  |  |  | 20 mA 4.30 hrs |
| **Promoter 5** | **F**-TCTATGGGAGGGGGAGACCT | 231 | 55 | 10% polyacryamide |
|  | **R**-GGAACTTGGTACAACCAGCTTG |  |  | 5% Glycerol |
|  |  |  |  | 20 mA 4.30 hrs |
| **Promoter 6** | **L**-CTGAACTCCCACTTGGCT | 208 | 55 | 10% polyacryamide |
|  | **R**-TGGAAGTGAGGAGGAGATG |  |  | 5% Glycerol |
|  |  |  |  | 20 mA 4.00 hrs |
| **Promoter 7** | **F**-GAGTACCAGGCTGTTGAGG | 195 | 55 | 10% polyacryamide |
|  | **R**-CACACTACTACGGACGGGA |  |  | 5% Glycerol |
|  |  |  |  | 20 mA 3.00 hrs |
| **Exon2-1** | **F**-TGTGTGTGGGGTCTGTCTCT | 265 | 55 | 10% polyacryamide |
|  | **R**-CCTTTCTCACCCTTCTCACC |  |  | 5% Glycerol |
|  |  |  |  | 20 mA 5.30 hrs |
| **Exon2-2** | **F**-GTGATGGCAGAGATGGCAC | 201 | 55 | 10% polyacryamide |
|  | **R**-CCAACCCCAAATCACTTCAG |  |  | 5% Glycerol |
|  |  |  |  | 20 mA 3.30 hrs |
| **Exon3-1** | **F**-GCCACAGGGATGGTAATT | 231 | 55 | 10% polyacryamide |
|  | **R**-TCTCCAATCCCACACTGA |  |  | 5% Glycerol |
|  |  |  |  | 20 mA 3.00 hrs |
| **Ex3-2** | **F**-GGAGAACCTGGAGAAGGTG | 249 | 55 | 10% polyacryamide |
|  | **R**-GAAGAGCATAGCCTTGTCCT |  |  | 5% Glycerol |
|  |  |  |  | 20 mA 4.30 hrs |
| **Ex3-3** | **F**-ATATGAAGGATGTGAAGGTC | 259 | 55 | 10% polyacryamide |
|  | **R**-AGGAGGCTCTGAGTTAGTG |  |  | 5% Glycerol |
|  |  |  |  | 20 mA 4.30 hrs |

All fragments were amplified by 40 cycles of (*i*) denaturation at 94°C for 30 seconds (5 minutes for the first cycle), (*ii*) annealing at temperatures indicated in the table for 30 seconds and (*iii*) extension at 72 °C for 30 seconds (5 minutes for the last cycle). These primers were also used for direct sequencing.
