# Supplementary material for: Novel Adiponectin Variants Identified in Type 2 Diabetic Patients Reveal Multimerization and Secretion Defects
Source: PLoS One. 2011 Oct 26;6(10):e26792. doi: 10.1371/journal.pone.0026792 (PMC3202584; doi:10.1371/journal.pone.0026792)
Supplement: Table S3 — Primers for amplification of ADIPOQ cDNA tagged with either FLAG or c-Myc sequences. (DOC) [file pone.0026792.s004.doc]

**Table S3 Primers for amplification of *ADIPOQ*** cDNA tagged with either FLAG or c-Myc sequences

| **Amplicon** | **Nucleotide sequence (5’🡪3’)** |
| --- | --- |
| **ADIPOQ-FLAG** | **F-**GGATCCATGCTGTTGCTGGGAGCTGTTCTCATAC |
|  | **R**-GGAATTCTCACTTATCGTCGTCATCCTTGTAATCGTTGGTGTCATGGTAGAGAAGAAAGCC |
| **ADIPOQ-Myc** | **F-** GGATCCATGCTGTTGCTGGGAGCTGTTCTCATAC |
|  | **R-** GGAATTCTCACAGATCCTCTTCTGAGATGAGTTTTTGTTCGTTGGTGTCATGGTAGAGAAGAAAGCC |
